# Nutritional and Phytochemical Variation of Marula (*Sclerocarya birrea*) (Subspecies *caffra* and *birrea*) Fruit among Nine International Provenances Tested in Malawi

**DOI:** 10.1155/2022/4686368

**Published:** 2022-10-11

**Authors:** Mussa Kamanula, Chimuleke Yagontha Munthali, John Finias Kamanula

**Affiliations:** ^1^Mzuzu University, Department of Forestry, Private bag 201, Luwinga, Mzuzu, Malawi; ^2^Mzuzu University, Department of Chemistry, Private bag 201, Luwinga, Mzuzu, Malawi

## Abstract

*Sclerocarya birrea* (Marula) is one of the indigenous fruit trees that was selected for domestication in Malawi. This study was conducted to assess nutritional and phytochemical variation of *Sclerocarya birrea* (subspecies *caffra* and *birrea*) fruits planted in an international provenance trial in Malawi. Vitamin C, calcium, iron, zinc, fat, and fibre content ranged from 6 to 81 mg/100 g; 1.8 to 5.3 mg/100 g; 1.4 to 3.3 mg/100 g; 0.3 to 0.8 mg/100 g; 51.6 to 57.7%; and 4.1 to 6.9%, respectively. Phytochemical scores showed that all nine provenances contained weak (+) concentration of alkaloids. Kalimbeza (Namibia) and Magamba-Turiani (Tanzania) provenances showed moderate (++) concentrations of saponins. Chikhwawa (Malawi), Missira (Mali), and Moamba (Mozambique) provenances had moderate (++) concentrations of tannins. Missira (Mali) and Kalimbeza (Namibia) provenances showed weak (+) concentration of terpenoids. Variations observed in nutritional and phytochemical composition could be attributed to genetic make-up and origin of the genotypes, since all genotypes were planted in the same environment. Therefore, selection of *Sclerocarya birrea* for domestication purposes should consider the provenance (origin of genotypes) and subspecies levels. Further studies should investigate vegetative propagation and heritability of nutritional and phytochemical traits before the use of seed for propagation.

## 1. Introduction

*Sclerocarya birrea* (Marula) is an indigenous fruit tree native to Southern Africa [1]. Its natural range spans northward through tropical Africa into Ethiopia and Sudan [2]. In West Africa, the tree is naturally found in Gambia, Nigeria, Cameroon, and Central African Republic [2]. In its native range, large populations of the tree are found in Namibia, Botswana, Zimbabwe, Zambia, Malawi, Swaziland, Mozambique, and South Africa [3]. Frost is the limiting factor affecting the distribution of *S. birrea* [4]. *Sclerocarya birrea* is a medium to large tree, usually 9 to 18 m tall and is single stemmed with short bore of up to 120 cm in diameter [5]. The tree grows in a wide range of soils but mostly prefers soils which are well drained [6]. It normally thrives in areas with altitudes varying from sea level to 1800 m with annual rainfall range of 200 to 1500 mm [7]. In Malawi, *S. birrea* has been reported to thrive in hot dry sites at 500 to 1000 m above sea level with mean annual rainfall and temperature of 900-1000 mm and 22–23°C, respectively [8].

*Sclerocarya birrea* is an indigenous fruit tree that is revered for its numerous socioeconomic contributions to human livelihood [9]. The female trees produce small mango-like edible fruits which have oil-bearing seeds [5]. The fruit pulp is widely consumed by humans as well as animals [5, 10]. Apart from raw consumption, the fruit pulp is used to make a variety of juices, jams, jellies, and liquor (Amarula) which are sold in national and international markets [11]. The fruit pulp has been reported to contain high levels of vitamin C (54 to194 mg/100 g) [12]. In addition, minerals such as calcium, iron, and zinc have been reported to occur in the fruit pulp [13]. The seed kernel is an important source of edible oil [5]. In addition, the seed kernel is used as a substitute for groundnuts in cooked vegetables especially in South Africa [14]. It is reported that the seed kernel is rich in fat and protein [11, 15]. Other nutritional components such as fibre and carbohydrates have also been reported in *S. birrea* seed kernel [16]. Various parts of *S. birrea* tree such as the root, leaves, bark, and seed kernel have, for a long time, been used for medicinal purposes [17, 18]. Extracts from the tree parts have acted as traditional remedies for treating diarrhoea, headache, toothache, stomachache, swollen legs, anaemia, malaria, high blood pressure, and scurvy [18, 19]. Several scientific examinations have also confirmed the availability of phytochemicals such as alkaloids, saponins, terpenoids, and tannins from the extracts of *S. birrea* tree parts [18, 20, 21]. Thus, apart from consumption, the fruit is also heavily exploited in the pharmaceutical industry.

*Sclerocarya birrea* is one of the indigenous fruit species that was selected for domestication in Southern Africa including Malawi to improve the nutritional status and well-being of rural communities through development of products for commercialization [22]. It has been argued that products from indigenous fruits such as *S. birrea* could contribute significantly to household nutrition and income [23]. However, it is hypothesized that the availability of *S. birrea* products could be achieved through domestication of the species [24]. In 1999, an international provenance trial for *S. birrea* was established in Malawi to drive the domestication of the species [25]. *Sclerocarya birrea* is one of the species whose populations have manifested large genetic variation for trees spanning large geographical distribution as well as geographically close individuals [26, 27]. However, the source and type of variation have been reported to determine the success of domestication programs [28]. In Malawi, studies on *S. birrea* in the international provenance trial have been done, showing variations in mating systems [29], pest susceptibility [30], growth performance, and fruit productivity [24] as well as fruit morphological traits within and among provenances [8]. However, information regarding nutritional and phytochemical variation of *S. birrea* in the trial is still outstanding. Consequently, it is not known whether or not the provenances are the same in terms of nutritional and phytochemical composition. Unearthing the nutritional and phytochemical composition of *S. birrea* could optimize the utilization and commercialization of the species and its products. In the present study, it is hypothesized that high variation exists in the nutritional and phytochemical composition of *S. birrea* populations collected from far wide geographic distribution. The present study was, therefore, carried out to assess nutritional and phytochemical variation that exist in *S. birrea* (subspecies *caffra* and *birrea*) genotypes planted in an international provenance trial in Mangochi, Malawi.

## 2. Methodology

### 2.1. Study Area and Experimental Design

**Figure 1:**
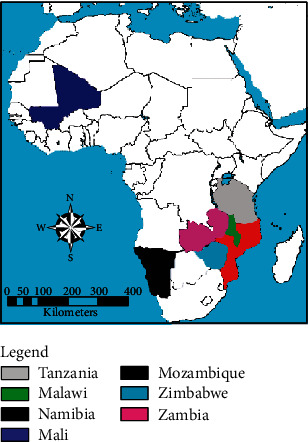
Countries where *Sclerocarya birrea* seed was collected.

Information of the study area is fully documented by [24, 25]. The trial was established in February 1999 in the Palm Forest Reserve in Mangochi, Malawi (14°28'S, 35°14'E). Palm Forest Reserve has an altitude of 200 m above sea level with mean annual rainfall of 800–1200 mm and mean annual temperature of 23.9°C. The area has flat terrain that consists of sandy soils and loamy sand with medium acidity. The seeds used in the trial were collected from provenances within SADC region (subspecies *caffra*) and Mali (subspecies *birrea*) (Figure 1). The experimental treatments were laid out as a randomized complete block design with 4 replicates. Each treatment had a line plot with twenty trees that represented the total possible number of families for each provenance. Spacing was five metres between row plots and four metres between trees within a plot, translating to eighty trees per population.

### 2.2. Collection of *Sclerocarya birrea* (Subspecies *caffra* and *birrea*) Fruits

**Figure 2:**
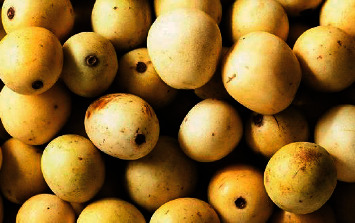
Phenotype of mature *Sclerocarya birrea* fruit.

Healthy, mature, and ripe fruits (Figure 2) for each provenance were collected from the ground (underneath trees) from January to December, 2019 (Table 1). It is reported that *S. birrea* fruits abscise before maturity and that ripening occurs on the ground [5]. Healthy fruits were the ones without pest damage, diseases, or any type of disorder. The ripe fruits were characterized by a thick yellow peel [5]. Fruits from each provenance were kept in a black polythene bag which was labelled. Fruits were kept in black polythene bags to prevent light from degrading vitamin C [31]. The fruits were then transported to Mzuzu University on the same day of collection.

### 2.3. Processing of *S. birrea* (Subspecies *caffra* and *birrea*) Fruit Pulp

**Figure 3:**
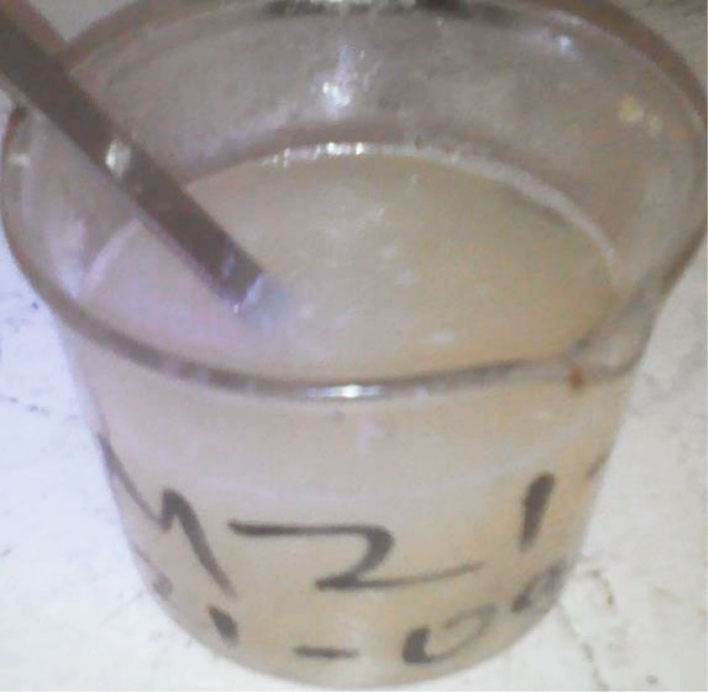
Appearance of *Sclerocarya birrea* fruit pulp.

*Sclerocarya birrea* (subspecies *caffra* and *birrea*) fruits were pressed by hands to obtain the fruit pulp. A sterilized blade was also used to peel off the remaining pulp which was attached to the hard-outer layer of the seed (endocarp). The fruit pulp was then placed in a dry clean beaker (Figure 3). The extraction of pulp was carried out in darkness to prevent loss of vitamin C [31].

### 2.4. Processing of *S. birrea* (Subspecies *caffra* and *birrea*) Seed Kernel

**Figure 4:**
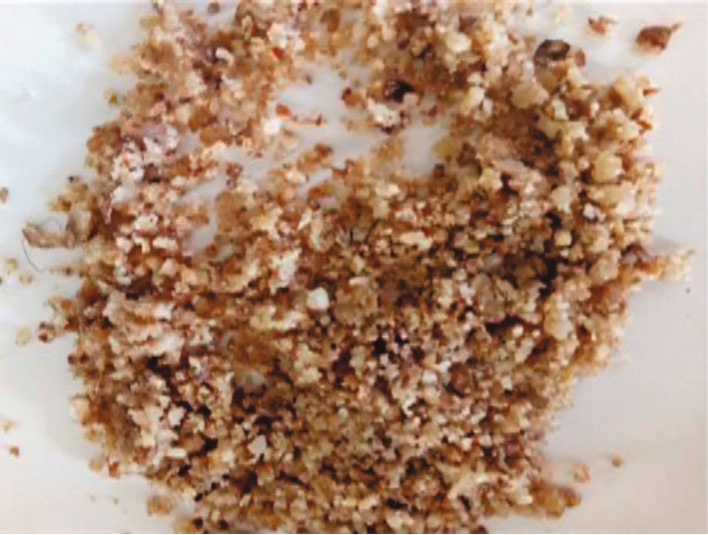
Pounded sample of *Sclerocarya birrea* seed kernel.

*Sclerocarya birrea* (subspecies *caffra* and *birrea*) seeds were obtained after removing the fruit pulp. The seeds were then dried (room temperature) under a shade for 7 days at Mzuzu University Chemistry Laboratory. The endocarps of the dried seeds were then crushed manually with a hammer to obtain the seed kernels. The seed kernels were dried under the shade for 5 to 7 days at room temperature until a constant weight was achieved. The kernels were then pounded using a wooden mortar and pestle. The pounded samples of the seed kernel (Figure 4) were then placed in clean plastic containers ready for nutritional and phytochemical analyses. Chemical analyses (nutrition and phytochemical) started immediately after preparation of the samples.

### 2.5. Determination of Moisture Content of *S. birrea* (Subspecies *caffra* and *birrea*) Fruit Pulp and Seed Kernel

A sample of fruit pulp (5 g) and seed kernel (5 g) were placed in separate dried and weighed crucibles in triplicate. The crucibles containing the fruit pulp and seed kernel were placed in an electric oven (Series 900; Bosch Electric Hobs, German) and were heated at a temperature of 100°C for 2 hours. The crucibles containing the pulp and seed kernel were then allowed to cool in a desiccator. After cooling, the crucibles were weighed on an analytical balance (N17250, Asynt, China). The moisture content was then expressed as a percentage ratio of weight lost to original weight of a sample [32].

### 2.6. Determination of Nutritional Composition of *S. birrea* (Subspecies *caffra* and *birrea*) Fruit Pulp

#### 2.6.1. Vitamin C

Vitamin C content of *S. birrea* (subspecies *caffra* and *birrea*) fruit pulp was determined using visual titration method employing the use of 2,6-dichloroindophenol dye [33]. An extract (20.00 mL) of fruit pulp+H_3_PO_4_:CH_3_COOH; 1 : 1 homogenized in an electric blender (Charles Ross & Son Company, USA) was placed into three Erlenmeyer flasks (250 mL) and was titrated to the end point using standardized 2,6-dichloroindophenol solution. A rose-pink colour that persisted for over 5 seconds indicated the titration end point. Three blank titrations were carried out using the reagents only. The amount of vitamin C present in *S. birrea* fruit pulp was then calculated using the following:

(1) $$\begin{array}{c} \text{Vitamin C }\left(\frac{\text{mg}}{100\text{g}}\right)=\left(X–B\right)*\left(\frac{F}{E}\right)*\left(\frac{V}{Y}\right)*100. \end{array}$$

*X* is the average sample titre volume (mL), *B* is the titre volume (mL) for the blank titration, *F* is mg ascorbic acid equivalent to 1.0 mL of indophenol solution, *E* is mass (g) of sample from which vitamin C was extracted, *V* is the volume (mL) of the initial assay solution, and *Y* is the volume (mL) of the aliquot used in titration.

#### 2.6.2. pH

The pH of *S. birrea* (subspecies *caffra* and *birrea*) fruit pulp was measured using a digital pH meter (Denver Instrument Basic model; Cole-Parmer, USA). The pH electrode was calibrated using two buffer solutions (pH 4.00 and pH 7.00, respectively). About 10 g of the fruit pulp was placed in a 200 mL glass beaker, and 100 mL of distilled water was added. The mixture was stirred thoroughly using an electric magnetic stirrer. The pH electrode was then dipped into the beaker containing the mixture, and the pH measurement was recorded when the reading had stabilized. The pH electrode was cleaned with tissue paper followed by distilled water before the next reading was taken. The measurements of pH were recorded in triplicate.

#### 2.6.3. Mineral Content (Calcium, Iron, and Zinc)

The amount of calcium, iron, and zinc present in *S. birrea* (subspecies *caffra* and *birrea*) fruit pulp was determined using atomic absorption spectrophotometer [34]. A dried sample (5 g) of *S. birrea* fruit pulp was placed in a 50 mL porcelain crucible in triplicate. The sample was then ashed by heating at a temperature of 650°C for 6 hours in an electric muffle furnace (CWF 1200; Bioevopeak, USA). After cooling, 7 mL of hydrochloric acid solution (6 M) were added to the ash and boiled on a hot plate with the aid of four antibumping granules until the solution had just dried. The crucible containing the sample was removed from the hot plate, and 10 mL hydrochloric acid solution (3 M) was added to the sample and boiled for 10 seconds on a hot plate. After cooling, the sample was filtered using Whatman filter paper No. 1 into a 100 mL volumetric flask. The filtered samples were finally diluted with distilled water up to the 100 mL mark. A blank sample which contained all reagents except the sample was treated in a similar manner as the samples. The sample solutions together with the blank were then taken to the Agricultural Research and Extension Trust (ARET) in Lilongwe (Malawi) for determination of metal ions using atomic absorption spectrophotometer (240FS; Agilent Technologies, German). From each metal calibration curve, the mineral content (calcium, iron, and zinc) was calculated using the following:

(2) $$\begin{array}{c} \text{Metal content }\left(\frac{\text{mg}}{100\text{g}}\right)=\left(a–b\right)*\left(\frac{V}{10W}\right). \end{array}$$

*W* is the weight (g) of the sample, *V* is the volume (mL) of extract (filtered sample), and *a* and *b* are concentrations (mg/L) of a sample solution and blank, respectively. These concentrations were determined from calibration curves for each metal.

### 2.7. Determination of Nutritional Composition of *S. birrea* (Subspecies *caffra* and *birrea*) Seed Kernel

#### 2.7.1. Fat Content

The amount of fat present in *S. birrea* (subspecies *caffra* and *birrea*) seed kernel was determined using the Soxhlet extraction method [35]. About 5 g of dried pounded sample of *S. birrea* seed kernel was weighed on an analytical balance (N17250, Asynt, China) and placed in an extraction thimble in triplicate. A piece of cotton wool was placed inside the extraction thimble to prevent loss of the sample. The extraction thimble was transferred into the Soxhlet extractor and 300 mL of n-hexane (analytical grade) was added. The Soxhlet extractor was attached to a weighed round bottomed flask which was placed on a heating mantle. The set-up was heated continuously for 6 hours and switched off and allowed to cool for 20 minutes. After extraction of fat, the solvent (n-hexane) was removed from the flask using a rotary evaporator (RE 111; BUCHI, Switzerland). The flasks were exposed to heat in an electric oven at 50°C for 5 minutes to eliminate further traces of the solvent. The amount of fat present in the sample was calculated using the following:

(3) $$\begin{array}{c} \text{Fat content }\left(\%\right)=\frac{\left(W3-W2\right)}{\left(W1\right)}*100. \end{array}$$

*W*1 is the weight (g) of sample before fat extraction, *W*2 is the weight (g) of flask without fat, and *W*3 is weight (g) of flask with fat.

#### 2.7.2. Fibre Content

The amount of fibre present in *S. birrea* (subspecies *caffra* and *birrea*) seed kernel was determined using a method developed by [36]. A dried grounded sample of *S. birrea* seed kernel (5 g) was placed in a 500 mL beaker, and 200 mL of 1.25% sulphuric acid was added. The mixture was boiled for 30 minutes and was later filtered by suction using a Buchner flask and funnel. The residue was then washed thoroughly with distilled water. Sodium hydroxide (200 mL of 1.25%, m/v) was added to the residue and the mixture was again boiled for 30 minutes. The mixture was then filtered by suction using a Buchner flask and funnel. The residue was thoroughly washed with distilled water and then rinsed with 10% hydrochloric acid, followed by 10% ethanol and finally with 10% petroleum ether. The residue was then placed in a crucible and heated in an electric oven (Series 900; Bosch Electric Hobs, German) at a temperature of 105°C for 24 hours. The crucible containing the residue was allowed to cool in a desiccator and was later transferred into a muffle furnace where it was ignited for 90 minutes at a temperature of 650°C to obtain the ash. The amount of fibre was calculated using the following:

(4) $$\begin{array}{c} \text{Fibre content }\left(\%\right)=\frac{\left(W2–W3\right)}{\left(W1\right)}*100. \end{array}$$

*W*1 is the weight (g) of the sample, *W*2 is the weight (g) of residue (insoluble matter before ashing), and *W*3 is the weight (g) of ash.

### 2.8. Phytochemical Screening of *S. birrea* (Subspecies *caffra* and *birrea*) Seed Kernel

#### 2.8.1. Test for Alkaloids

Screening for alkaloids was carried out following a method by [36]. A dried pounded sample (5 g) of *S. birrea* seed kernel was macerated in 50 mL of 5% (v/v) hydrochloric acid solution. The mixture was stirred for 5 minutes using a glass rod. The mixture was then left to stand for 24 hours. After 24 hours, the mixture was filtered using a suction machine. A portion of the filtrate (1 mL) was placed in a test tube and 10 drops of Dragendorff's reagent were added to the filtrate. Presence of a red or orange precipitate indicated the presence of alkaloids.

#### 2.8.2. Test for Saponins

Test for saponins was carried out using a method described by [37]. An infusion (5% w/v) was prepared by macerating 1 g of dried pounded sample of *S. birrea* seed kernel in 20 mL of distilled water. The mixture was stirred for 5 minutes and was left to stand for 24 hours. The extract was then filtered using Whatman filter paper No.1. A portion of the filtrate (10 mL) was transferred into a test tube and was shaken vigorously for 10 seconds. The length of the foam that persisted for 10 minutes was measured using a ruler (cm) and used as an indication for the presence of saponins.

#### 2.8.3. Test for Tannins

Test for tannins was done using a method by Harborne [36]. An infusion (5% w/v) was prepared by macerating 1 g of dried pounded sample of *S. birrea* seed kernel in 20 mL of distilled water then stirred for about 5 minutes. The mixture was left to stand for 24 hours. The extract was then filtered with a suction machine. The filtrate (2 mL) was poured into a test tube and 10 drops of 0.5 M Ferric chloride aqueous solution were added. Blue, black, or green precipitates indicated the presence of tannins.

#### 2.8.4. Test for Terpenoids

Test for terpenoids was done using a method by [36]. A dried pounded sample of *S. birrea* seed kernel (1 g) was macerated in 20 mL of diethyl ether in a stoppered conical flask. The mixture was left to stand for 48 hours. The extract was then filtered using Whatman filter paper No.1. A portion of the filtrate (1 mL) was placed in a porcelain crucible and was dried on a hot plate followed by the addition of 10 drops of concentrated sulphuric acid. The colour that was produced in the mixture was recorded. Another fresh portion of the sample filtrate (1 mL) was treated with 1 mL of acetic anhydride followed by 1 mL of concentrated sulphuric acid. The appearance of green, blue, and pink to purple colours in the mixture indicated the presence of terpenoids.

### 2.9. Data Analysis

Measurements of vitamin C, pH, calcium, and iron were tested for normality and homogeneity with the Kolmogorov-Smirnov test using Minitab 17. Values of fat (%) and fibre content (%) were subjected to arcsine transformation using Microsoft Excel 2013. After meeting the above criteria, data were subjected to one-way analysis of variance (*P* ≤ 0.05) using Minitab 17. Significantly different mean values were separated using Fisher's Least Significant Difference (LSD). Values of zinc content were analysed using descriptive statistics. Variance percentage for zinc content was used to illustrate the magnitude of variation for the parameter between the lowest and highest interval (Table 2). The notation (np) meant that the parameter (zinc) was not present in a particular provenance. The concentrations of phytochemicals (alkaloids, saponins, tannins, and terpenoids) were assessed using qualitative scores (+++, ++, +, and −) where +++ denoted strong concentration, ++ represented moderate concentration, + indicated weak concentration, and − indicated absence of a phytochemical.

## 3. Results

Results presented in this study are based on dry matter basis. The moisture content of the fruit pulp ranged from 80.4 to 80.9% while that of seed kernel ranged from 4.1 to 4.2%.

### 3.1. Nutritional Variation of *S. birrea* (Subspecies *caffra* and *birrea*) Fruit Pulp and Seed Kernel

A summary of nutritional variation of *S. birrea* (subspecies *caffra* and *birrea*) fruit pulp and seed kernel is presented in Table 2. There were variations in nutritional composition of *S. birrea* fruit pulp (vitamin C, Ca, Fe, and Zn) and seed kernel (fat and fibre content) among the provenances. The highest variation (9500% variance) was generally observed in the amount of vitamin C content among the nine provenances.

### 3.2. Nutritional Composition of *S. birrea* (Subspecies *caffra* and *birrea*) Fruit Pulp

Table 3 shows the results of vitamin C, pH, and mineral content (calcium, iron, and zinc) of *S. birrea* (subspecies *caffra* and *birrea*) fruit pulp among nine provenances.

#### 3.2.1. Vitamin C

There were significant differences (*P* ≤ 0.05) in the amount of vitamin C of *S. birrea* (subspecies *caffra* and *birrea*) fruit pulp among the nine provenances. Vitamin C was highest in Ngundu provenance (81 ± 3.2 mg/100 g) (subspecies *caffra*), followed by Marracuene (48 ± 9.4 mg/100 g) (subspecies *caffra*), and Moamba (48 ± 1.4 mg/100 g) (subspecies *caffra*). The third highest values of vitamin C were from Matebeleland (35 ± 2.5 mg/100 g) (subspecies *caffra*) and Missira (30 ± 0.3 mg/100 g) (subspecies *birrea*), followed by Magunde (23 ± 3.3 mg/100 g) (subspecies *caffra*). The lowest contents were for populations of subspecies *caffra* from Magamba-Turiana (12 ± 0.2 mg/100 g), Kalimbeza (10 ± 1.1 mg/100 g), and Chikhwawa (6 ± 0.2 mg/100 g).

#### 3.2.2. pH

The pH of *S. birrea* (subspecies *caffra* and *birrea*) fruit pulp was significantly different (*P* ≤ 0.05) among the provenances, and two groups were noted. The highest pH was observed in populations from Chikhwawa (4.17 ± 0.02) (subspecies *caffra*), Moamba (4.03 ± 0.1) (subspecies *caffra*), Ngundu (3.95 ± 0.02) (subspecies *caffra*), Magunde (3.95 ± 0.07) (subspecies *caffra*), Magamba-Turiana (3.93 ± 0.08) (subspecies *caffra*), Marracuene (3.90 ± 0.1) (subspecies *caffra*), Matebeleland (3.76 ± 0.07) (subspecies *caffra*), and Missira (3.88 ± 0.1) (subspecies *birrea*). The lowest pH (3.07 ± 0.01) was in Kalimbeza population (subspecies *caffra*).

#### 3.2.3. Calcium (Ca)

Significant differences (*P* ≤ 0.05) were observed in the calcium content of *S. birrea* (subspecies *caffra* and *birrea*) fruit pulp among the nine provenances. The highest amount (5.3 ± 0.04 mg/100 g) was in Kalimbeza provenance (subspecies *caffra*) followed by Missira (4.5 ± 0.8 mg/100 g) (subspecies *birrea*), Chikhwawa (4.2 ± 0.03 mg/100 g) (subspecies *caffra*)), Marracuene (4.0 ± 0.4 mg/100 g) (subspecies *caffra*), Magunde (3.8 ± 0.03 mg/100 g) (subspecies *caffra*), and Matebeleland (3.7 ± 0.06 mg/100 g) (subspecies *caffra*). The lowest content was in Moamba provenance (2.6 ± 0.4 mg/100 g) (subspecies *caffra*), Magamba-Turiani (2.4 ± 0.02 mg/100 g) (subspecies *caffra*), and Ngundu (1.8 ± 0.02 mg/100 g) (subspecies *caffra*).

#### 3.2.4. Iron (Fe)

Iron content was significantly different (*P* ≤ 0.05) in *S. birrea* (subspecies *caffra* and *birrea*) fruit pulp from the nine provenances. Iron content ranged from 1.4 to 3.3 mg/100 g. The highest amount was found in Chikhwawa (3.3 ± 0.1 mg/100 g) (subspecies *caffra*) and Marracuene (3.3 ± 0.5 mg/100 g) (subspecies *caffra*) followed by Missira (2.8 ± 0.4 mg/100 g) (subspecies *birrea*), Kalimbeza (2.6 ± 0.1 mg/100 g) (subspecies *caffra*), Magamba-Turiani (2.1 ± 0.1 mg/100 g) (subspecies *caffra*), and Matebeleland (2.0 ± 0.04 mg/100 g) (subspecies *caffra*). The lowest iron content (1.4 ± 0.07 mg/100 g) was found in Magunde provenance (subspecies *caffra*).

#### 3.2.5. Zinc (Zn)

Zinc content ranged from 0.3 ± 0.01 mg/100 g for Chikhwawa (subspecies *caffra*) to 0.8 ± 0.02 mg/100 g for Ngundu provenance (subspecies *caffra*), representing 14% variance (Table 3). Marracuene and Matebeleland provenances had 0.06 mg/100 g of zinc in their fruit pulp. Zinc was absent or not present (np) in five provenances namely Magamba-Turiani (subspecies *caffra*), Moamba (subspecies *caffra*), Magunde (subspecies *caffra*), Kalimbeza (subspecies *caffra*), and Missira (subspecies *birrea*).

### 3.3. Nutritional Composition of *S. birrea* (Subspecies *caffra* and *birrea*) Seed Kernel

Results of fat and fibre content of *S. birrea* (subspecies *caffra* and *birrea*) seed kernel are shown in Table 4.

#### 3.3.1. Fat Content

There were significant differences (*P* ≤ 0.05) in the amount of fat available in *S. birrea* (subspecies *caffra* and *birrea*) seed kernel among the nine provenances. Fat content ranged from 51 to 57%. The highest fat content (57.7 ± 0.1%) was found in Missira provenance (subspecies *birrea*) which was followed by Kalimbeza (55.1 ± 0.1%) (subspecies *caffra*). The lowest fat content was found in populations of subspecies *caffra* from Chikhwawa (52.9 ± 0.2%), Marracuene (51.9 ± 0.1%), Magunde (51.7 ± 0.2%), Moamba (52.9 ± 0.2%), Matebeleland (52.5 ± 0.2%), Ngundu (51.6 ± 0.1%), and Magamba-Turiani (51.7 ± 0.2%).

#### 3.3.2. Fibre Content

Fibre content was significantly different (*P* ≤ 0.05) among the nine provenances. The highest content (6.9 ± 0.01%) was in Chikhwawa (subspecies *caffra*) followed by Matebeleland (6.1 ± 0.01%) (subspecies *caffra*). The lowest fibre content was observed in Missira (4.8 ± 0.01%) (subspecies *birrea*), Marracuene (4.6 ± 0.06%) (subspecies *caffra*), Magunde (4.5 ± 0.1%) (subspecies *caffra*), Moamba (4.4 ± 0.07%) (subspecies *caffra*), Ngundu (4.4 ± 0.1%) (subspecies *caffra*), Magamba-Turiani (4.3 ± 0.06%) (subspecies *caffra*), and Kalimbeza (4.1 ± 0.01%) (subspecies *caffra*).

### 3.4. Phytochemical Screening of *S. birrea* (Subspecies *caffra* and *birrea*) Seed Kernel

Table 5 shows the phytochemical composition of *S. birrea* (subspecies *caffra* and *birrea*) seed kernel among nine provenances. The concentration of alkaloids was the same in all the nine sampled provenances. All provenances showed weak (+) concentration of alkaloids. For saponins, seven populations namely Chikhwawa (subspecies *caffra*), Marracuene (subspecies *caffra*), Moamba (subspecies *caffra*), Ngundu (subspecies *caffra*), Matebeleland (subspecies *caffra*), Magunde (subspecies *caffra*), and Missira (subspecies *birrea*) had weak (+) concentrations, while two populations from Kalimbeza (subspecies *caffra*) and Magamba-Turiani (subspecies *caffra*) had moderate (++) concentrations. For tannins, six populations from Marracuene (subspecies *caffra*), Magunde (subspecies *caffra*), Matebeleland (subspecies *caffra*), Ngundu (subspecies *caffra*), Magamba-Turiani (subspecies *caffra*), and Kalimbeza (subspecies *caffra*) had weak concentrations, while three populations from Missira (subspecies *birrea*), Chikhwawa (subspecies *caffra*), and Moamba (subspecies *caffra*) had moderate (++) concentrations. For terpenoids, only two populations from Kalimbeza (subspecies *caffra*) and Missira (subspecies *birrea*) had weak (+) concentrations, while the remaining seven populations did not contain the phytochemical.

## 4. Discussion

### 4.1. Nutritional Composition of *S. birrea* (Subspecies *caffra* and *birrea*) Fruit Pulp and Seed Kernel

Nutritional composition of *S. birrea* (subspecies *caffra* and *birrea*) fruit pulp and seed kernel has shown to be variable among the nine provenances. Nutritional differences are influenced by the genetic make-up of species [38] and environmental variation [26]. In the present study, nutritional differences observed in *S. birrea* seed kernel could be attributed to genetic differences or origin of populations (provenances), since all the genotypes were grown in the same environment. Thus, the genotypes were exposed to similar soil type, temperature, humidity, rainfall, light intensity, and other environmental variables.

### 4.2. Nutritional Composition of *S. birrea* (Subspecies *caffra* and *birrea*) Fruit Pulp

#### 4.2.1. Vitamin C

The high variance percentage (9500%) in vitamin C (Table 2) clearly shows the large variation that exists in the amount of vitamin C among *S. birrea* populations from different provenances. This large variation shows that there are specific populations within the species with high vitamin C content and these populations could be selected and incorporated in domestication and breeding programs. In addition, the large variation shows the possibility of attaining high genetic gains through selection at provenance and subspecies levels. The high value of vitamin C (81 mg/100 g) (Ngundu provenance; subspecies *caffra*) is lower than the reported value of vitamin C of *S. birrea* fruit pulp from Kenya (190 mg/100 g) [13] but higher than that of orange (41 mg/100 g), lemon (23 mg/100 g), pawpaw (39 mg/100 g), and pineapple (38 mg/100 g) [39]. Vitamin C is an important product for treating scurvy [40] and reducing the incidence of cancer [41]. The vitamin C content of *S. birrea* fruit pulp, therefore, makes it an important product that could treat scurvy and reduce the incidence of cancer. Thus, the fruit pulp could form an important diet in most rural areas of Malawi where scurvy is a challenge once the species gets promoted. Further research should test on genotype and environmental interaction and heritability of the trait (vitamin C).

#### 4.2.2. pH

The fruit pulp of *S. birrea* (subspecies *caffra* and *birrea*) from all the nine provenances were acidic with pH values ranging from 3.07 to 4.17 (Table 3). These values are comparable to the pH levels of lemon (3.1), orange (4.0), and grape (3.2) pulp [42]. The low pH (high acidity) of *S. birrea* fruit pulp could be attributed to the presence of vitamin C. It is reported that vitamin C (ascorbic acid) is slightly acidic, and that significant amounts of it can lower the pH of a substance [43]. The pH levels below 4.6 have been reported to hinder the production of toxins from microorganisms during food storage, thereby increasing the product's shelf life [44]. The low pH of *S. birrea* fruit pulp (3.07 to 4.17) could, therefore, be beneficial during preservation or storage of the pulp or products emanating from the pulp. Probably, *S. birrea* fruit pulp could be blended with other foodstuffs to produce final products with a considerable shelf life. On the other hand, food products with high acidity (low pH) have been reported to cause damage of the enamel layer of the teeth [45]. Consumption of *S. birrea* fruit pulp in large quantities could, therefore, instigate tooth wear. Perhaps, the fruit pulp could also be blended with other food products to yield an end product with a much desired pH.

#### 4.2.3. Calcium

Calcium content represents a 68% variance among the study populations (Table 2). As mentioned earlier, the variation could be caused by genetic differences and origin of genotypes (provenance), since the populations were grown in the same environment. This variation suggests that there is a possibility of selecting provenances with significant quantities of calcium at provenance and subspecies levels. The range of calcium (1.8 mg/100 g to 5.3 mg/100 g) found in this study is lower than the calcium content (310 mg/100 g) recorded in *S. birrea* fruit pulp from Burkina Faso [46]. The large variation in calcium content could be attributed to genetic or environmental differences [47]. Calcium is an important element that is essential in the formation of strong bones and teeth [48]. The presence of calcium suggests that *S. birrea* fruit pulp could form a significant diet to aid the formation of strong bones and teeth for the majority of minors and elder people in Malawi once the species is promoted and commercialized.

#### 4.2.4. Iron

Iron content ranged from 1.4 to 3.3 mg/100 g representing a 33% variance (Table 2). This variation also provides a chance of selecting populations in breeding/domestication programs. The highest value of iron (3.3 mg/100 g) found in this study is slightly higher than the iron content of *S. birrea* fruit pulp reported in Kenya (2.7 mg/100 g) [13] but lower than the commended daily intake of 18 mg/day [49]. The variation in iron content could be attributed to genetic make-up of species [50] and environmental variation [26]. Iron is an important element that improves the levels of haemoglobin and aids the oxidation of fats, proteins, and carbohydrates [51]. The populations tested in the present study could supplement iron to improve the levels of haemoglobin and help the oxidation of fats, proteins, and carbohydrates in the human body. The fruit pulp could also become an important diet in pregnant women who require adequate blood supply.

#### 4.2.5. Zinc

The amount of zinc (0.3 to 0.8 mg/100 g) as a trace element was small but in substantial amount (Table 3). The range of zinc content represents a 14% variance (Table 2). The values found in the present study are higher than that reported in a study conducted in Botswana [52] who found 0.13 mg/100 g of zinc in *S. birrea* fruit pulp. This variation in zinc content could be due to both environmental and genetic differences [50, 53]. Prasad [54] noted that zinc deficiency in pregnant women leads to impairment of zygotes. Thus, *S. birrea* fruit pulp could form an important diet in pregnant women with zinc deficiency. It is reported that zinc is an essential element that is involved in digestion and in the release of hormones [50] and nerve impulses [55]. Therefore, the presence of zinc suggests that *S. birrea* fruit pulp could also form a significant diet at various stages of human development as the fruit is eaten raw. Currently in Malawi, the species is not taken seriously and yet products from the fruit pulp may greatly improve the livelihood of both rural and urban people through consumption of fruits and fruit products.

### 4.3. Nutritional Composition of *S. birrea* (Subspecies *caffra* and *birrea*) Seed Kernel

#### 4.3.1. Fat Content

The highest fat content (57.7%) was for Missira provenance (subspecies *birrea*) (Table 4) which is comparable to that of *S. birrea* subspecies *caffra* from Kenya (57.3%) [13] and Ghana (57.2%) [16]. FAO [56] reported that fats are essential components of energy in the human body. Consumption of *S. birrea* seed kernel could, therefore, provide energy to the human body. The American Council on Exercise [57] noted that fats are vital in nerve impulse transmission and hormone production. Therefore, *S. birrea* seed kernel could be incorporated in diets as a source of fat to aid nerve impulse transmission and hormone production for the prevention of neurodegenerative diseases. Future research could further explore the nutritional characteristics of fats extracted from *S. birrea* seed kernel. In addition, further studies should investigate on the heritability of the trait in subspecies *birrea* having the greatest amount of fat.

#### 4.3.2. Fibre Content

The fibre content ranged from 4.1 to 6.9% representing 22% variance (Table 4). These values are higher than the fibre content of *S. birrea* seed kernel from Ghana (2.4%) [16] and that of maize (2.1%) [58]. The variation could be attributed to genetic differences [40] and environmental variation [26]. Fibre is a dietary component that is essential in the prevention of diabetes, colon cancer, and constipation [59]. Furthermore, high levels of fibre content reduce the amount of serum cholesterol and minimize the risk of coronary heart diseases [60]. The consumption of *S. birrea* seed kernel could, therefore, help to prevent diabetes, colon cancer, constipation, and coronary heart diseases. The fibre in *S. birrea* could also act as a substitute for fibre available in maize which is a staple food in Malawi. Moreover, the Malawian genotype (Chikhwawa) showed the highest fibre content among the nine provenances.

### 4.4. Phytochemical Composition of *S. birrea* (Subspecies *caffra* and *birrea*) Seed Kernel

The results of the study (Table 5) have revealed the occurrence of weak (+) concentration of alkaloids, weak (+) to moderate (++) concentration of saponins, weak (+) to moderate (++) concentration of tannins, and absent (−) to weak (+) concentration of terpenoids in *S. birrea* (subspecies *caffra* and *birrea*) seed kernel originating from the nine provenances. Differences in levels of phytochemicals have been reported to be influenced by genetic and environmental factors [50, 61]. Phytochemical variations observed in *S. birrea* seed kernel could, therefore, be due to genetic differences since all populations/provenances were grown in the same environment. These variations could be of great importance during the selection of populations in breeding and domestication programs. In addition, the presence of different phytochemicals at provenance level shows the possibility of different pharmacological applications of *S. birrea* seed kernel.

#### 4.4.1. Alkaloids

The weak (+) concentration of alkaloids in *S. birrea* (subspecies *caffra* and *birrea*) seed kernel in all provenances indicate that the occurrence of this phytochemical is the same among provenances as well as subspecies. In other studies, [62, 63] have also demonstrated the presence of alkaloids in *S. birrea* seed kernel. The alkaloids have been reported to possess analgesic (pain killing) properties [63]. The presence of alkaloids, therefore, suggests that *S. birrea* seed kernel could be a raw material to synthesize alkaloid pain killers. In other studies, alkaloids have displayed antimalarial properties [64]. This, therefore, indicate that *S. birrea* seed kernel containing alkaloids could form traditional antimalarial remedies. In Malawi, most rural dwellers largely depend on traditional remedies, as such species could become an important herbal therapy.

#### 4.4.2. Saponins

Phytochemical scores revealed weak (+) to moderate (++) concentrations of saponins in *S. birrea* seed kernel originating from different provenances. Some researchers [65] have reported that saponins control blood sugar levels in the human body. Consumption of *S. birrea* seed kernel with substantial amount of saponins could help to reduce the risk of diabetes. It has also been noted that saponins display anticancer properties [66]. The consumption of *S. birrea* seed kernel containing saponins could reduce the risk of developing cancer. Mohammed et al. [21] further reported the antibacterial properties of saponins extracted from *S. birrea* root, stem, and leaves, specifically against *Escherichia coli*. Probably, extracts of *S. birrea* seed kernel containing saponins could also display antibacterial properties against *Escherichia coli*. Populations such as Magamba-Turiani and Kalimbeza (subspecies *caffra*) could form ideotypes for production of *S. birrea* seed kernel with moderate (++) levels of saponins. Future research could focus on vegetative propagation of the trees to maintain moderate levels of saponins.

#### 4.4.3. Tannins

The concentration of tannins in *S. birrea* seed kernel ranged from weak (+) to moderate (++). Such variations could also be attributed to genetic differences associated with origin of genotypes since all provenances were grown in the same environment. Several researchers [21, 52] have reported the antibacterial activity of saponins extracted from *S. birrea* stem bark, root, and leaves. Perhaps, extracts of *S. birrea* seed kernel containing tannins could also display antimicrobial activity. Probably, the raw seed kernel could even be easier to swallow than extracts of root, leaves, and stem bark. Ukoha et al. [67] acknowledged the antiseptic properties of tannins. The presence of tannins, therefore, suggests that *S. birrea* seed kernel could be used as a treatment for skin irritations and wounds. Furthermore, tannins have been reported to possess anticancer properties [68]. Consumption of *S. birrea* seed kernel rich in tannins could, hence, reduce the probability of developing cancer. Provenances like Chikhwawa, Moamba, and Missira could also be selected to produce ideotypes with moderate (++) concentrations of tannins.

#### 4.4.4. Terpenoids

The study has revealed the occurrence of absent (−) to weak (+) concentrations of terpenoids among the nine provenances of *Sclerocarya birrea* being screened in Malawi. Missira and Kalimbeza provenances showed weak (+) concentration of terpenoids while the rest of the provenances did not contain terpenoids (−). In Burkina Faso, [18] also noted the presence of terpenoids in *S. birrea* seed kernel. Mohammed et al. [21] reported the antimicrobial activity of terpenoids extracted from *S. birrea* leaf and root. Perhaps, extracts of *S. birrea* seed kernel containing terpenoids could also exhibit antimicrobial activity. In another study, [69] reported that terpenoids possess antifungal properties. The availability of terpenoids indicates that seed kernels of trees from Missira and Kalimbeza provenances could be used to fight fungal infections. It is further reported that terpenoids are antitumor agents [70]. Therefore, the presence of terpenoids indicates that seed kernels of trees from Missira and Kalimbeza provenances could be used to treat tumors. Most importantly, [71] acknowledged that terpenoids are precursors of sex hormones such as testosterone. *Sclerocarya birrea* seed kernels containing terpenoids could, therefore, help to improve the reproductive status of males with low counts of testosterone. For commercial purposes, *S. birrea* seed kernel could possibly be processed into tablets, syrups, or capsules to treat bacterial and fungal diseases and to supplement levels of testosterone in men with low counts of the hormone.

## 5. Conclusions

The study is aimed at assessing the nutritional and phytochemical variation of *Sclerocarya birrea* (subspecies *caffra* and *birrea*) fruit among nine international provenances being screened in Malawi. Results have revealed that there are significant variations in nutritional and phytochemical composition of *S. birrea* (subspecies *caffra* and *birrea*) fruit among populations collected from different provenances in Africa. These variations could be attributed to genetic make-up of the populations or origin of the populations (provenances). Therefore, the selection of *S. birrea* in domestication or breeding programs must reflect on both provenance and subspecies levels if target ideotypes are to be developed. Additional research should examine heritability of important nutritional and phytochemical traits before conclusive decisions on the use of seed for propagation are carried out.

## Figures and Tables

**Table 1:** Sampling time of *Sclerocarya birrea* populations (countries and provenances).

Country	Provenance	Subspecies	Maturity time
Malawi	Chikhwawa	*caffra*	April
Mozambique	Marracuene	*caffra*	December
Mozambique	Magunde	*caffra*	January
Mozambique	Moamba	*caffra*	December
Mali	Missira	*birrea*	November
Zimbabwe	Matebeleland	*caffra*	January
Zimbabwe	Ngundu	*caffra*	January
Namibia	Kalimbeza	*caffra*	April
Tanzania	Magamba-Turiani	*caffra*	April

**Table 2:** Variation in nutrition composition of *S. birrea* fruit pulp and seed kernel.

Parameter	Mean	Interval	Variance	Variance%
Vitamin C	32.5 mg/100 g	6.0 to 81.0	570	9500
Ca	3.58 mg/100 g	1.8 to 5.3	1.24	68
Fe	2.34 mg/100 g	1.4 to 3.3	0.47	33
Zn	0.57 mg/100 g	0.3 to 0.8	0.043	14
Fat	53.10%	51.6 to 57.7	4.15	8
Fibre	4.90%	4.1 to 6.9	0.9	22

**Table 3:** Nutritional composition (vitamin C, pH, Ca, Fe, and Zn) of *S. birrea* fruit pulp among nine provenances.

Provenance	Vit C (mg/100 g)	pH	Mineral content (mg/100 g)		
			Ca	Fe	Zn
Chikhwawa (*caffra*)	6 ± 0.2^f^	4.17 ± .02^a^	4.2 ± .03^b^	3.3 ± 0.1^a^	0.3 ± .01
Marracuene (*caffra*)	48 ± 9.4^b^	3.90 ± 0.1^a^	4.0 ± 0.4^b^	3.3 ± 0.5^a^	0.6 ± 0.1
Magunde (*caffra*)	23 ± 3.3^d^	3.95 ± .07^a^	3.8 ± .03^bc^	1.4 ± .07^c^	Np
Moamba (*caffra*)	48 ± 1.4^b^	4.03 ± 0.1^a^	2.6 ± 0.4^de^	1.9 ± .06^c^	Np
Matebeleland (*caffra*)	35 ± 2.5^c^	3.76 ± .07^a^	3.7 ± .06^bc^	2.0 ± .04	0.6 ± .02
Ngundu (*caffra*)	81 ± 3.2^a^	3.95 ± .01^a^	1.8 ± .02^e^	1.7 ± .07^c^	0.8 ± .02
Magamba-Turiani (*caffra*)	12 ± 0.2^ef^	3.93 ± .08^a^	2.4 ± .02^de^	2.1 ± 0.1^b^	Np
Kalimbeza (*caffra*)	10 ± 1.1^ef^	3.07 ± .01^b^	5.3 ± .04^a^	2.6 ± 0.1^b^	Np
Missira (*birrea*)	30 ± 0.3^c^	3.88 ± 0.1^a^	4.5 ± 0.8^b^	2.8 ± 0.4^b^	Np

^∗^Means which do not share a letter within a column are significantly different (*P* ≤ 0.05). ^∗^Means are followed by standard error; Vit C stands for vitamin C; np denotes “not present”.

**Table 4:** Nutritional composition of *S. birrea* (subspecies *caffra* and *birrea*) seed kernel among nine provenances.

Provenance	Subspecies	Fat content (%)	Fibre content (%)
Chikhwawa	*caffra*	52.9 ± 0.2^c^	6.9 ± .01^a^
Marracuene	*caffra*	51.9 ± 0.1^c^	4.6 ± .06^c^
Magunde	*caffra*	51.7 ± 0.2^c^	4.5 ± 0.1^c^
Moamba	*caffra*	52.9 ± 0.2^c^	4.4 ± .07^c^
Matebeleland	*caffra*	52.5 ± 0.2^c^	6.1 ± .01^b^
Ngundu	*caffra*	51.6 ± 0.1^c^	4.4 ± 0.1^c^
Magamba-Turiani	*caffra*	51.7 ± 0.2^c^	4.3 ± .06^c^
Kalimbeza	*caffra*	55.1 ± 0.1^b^	4.1 ± .01^cd^
Missira	*birrea*	57.7 ± 0.1^a^	4.8 ± .01^c^

^∗^Means which do not share a letter within a column are statistically different (*P* ≤ 0.05). ^∗^Mean values are followed by standard error.

**Table 5:** Phytochemical composition of *S. birrea* (subspecies *caffra* and *birrea*) seed kernel between provenances.

Provenance	Subspecies	Alkaloids	Phytochemical		
			Saponins	Tannins	Terpenoids
Chikhwawa	*caffra*	+	+	++	−
Marracuene	*caffra*	+	+	+	−
Magunde	*caffra*	+	+	+	−
Moamba	*caffra*	+	+	++	−
Matebeleland	*caffra*	+	+	+	−
Ngundu	*caffra*	+	+	+	−
Magamba-Turiani	*caffra*	+	++	+	−
Kalimbeza	*caffra*	+	++	+	+
Missira	*birrea*	+	+	++	+

^∗^Concentrations (+++=high, ++=moderate, +=weak, and −=absent).

## Data Availability

The data used to support the findings of this study are available from the corresponding author upon request.
